# A Non-Isokinetic Approach for Modeling Solid-State Transformations: Application to Crystallization of a Fe-B Amorphous Alloy

**DOI:** 10.3390/ma14020292

**Published:** 2021-01-08

**Authors:** Yazhu Ma, Yubing Zhang, Feng Liu

**Affiliations:** State Key Laboratory of Solidification Processing, Northwestern Polytechnical University, Xi’an 710072, Shaanxi, China; yazhu.ma@gmx.de (Y.M.); zyb@mail.nwpu.edu.cn (Y.Z.)

**Keywords:** isochronal transformation, non-isokinetic, crystallization, Fe-B alloy

## Abstract

Solid-state phase transformations like crystallization of amorphous alloys can be described by an analytical model incorporating a nucleation index *a*. However, this model cannot be used to examine isochronal transformations with abrupt changing of enthalpy differences performed with differential scanning calorimetry. Based on the model, a non-isokinetic approach is proposed and applied to analyze the isochronal crystallization kinetics of Fe_85_B_15_ amorphous alloy. The approach enabled us to obtain the kinetic parameters and activation energies for nucleation and growth.

## 1. Introduction

Crystallization is usually studied using Johnson–Mehl–Avrami (JMA) kinetics [1,2,3,4,5,6,7,8,9,10]. The classical JMA equation is applied in the kinetic analysis since constant kinetic parameters can be applied to study a series of isothermal transformations carried out at different temperatures [5,6] or isochronal transformations carried out with varying rates of heating [7,8,9,10]. Kinetic model parameters and impingement mode are not relevant to the annealing temperature for isothermal transformations, or to the heating rate Φ for isochronal transformations. The model parameters including the rate constant *K*_0_, the Avrami/growth exponent *n* and the effective activation energy *Q* are constant by using the JMA equation [5,6,10]. Isokinetics means that the mechanisms of nucleation, growth and impingement are constant upon transformations, although, in general, the isokinetic approximation does not hold in the framework of the JMA model [11,12,13,14].

An analytical phase transformation model was proposed by Liu et al. [15,16,17,18,19,20,21,22,23,24], where nucleation, growth mechanisms and impingent mode are integrated. The model has been used to interpret various solid-state transformations like crystallization of amorphous alloys [16,17,18,23] and is deduced within the framework of the JMA equation [15,16,17,18,19], but the kinetic parameters *n*, *Q*, *K*_0_ depend on time for isothermal transformations, and on temperature for isochronal transformations. Both are affected by parameters such as the nucleation and growth activation energy, *Q_N_* and *Q_G_*. For a case where the nucleation rate increases with crystallization progress, a nucleation index *a* [19,25,26] is introduced into the analytical transformation model. For *a* > 1, the nucleation rate does not remain constant but increases throughout the transformation [27,28,29,30].

The analytical model is based on the extended isokinetic theory. The mechanism (such as nucleation, growth and impingement) stays the same as transformation progresses, despite the change of the model parameters. As such, *n* changes as a function of temperature [25] (for the isothermal case) or of heating rate [31,32] (for the isochronal case). With a constant *a* (≥1), the growth exponent *n* (*d*/*m* < *n* < *d*/*m* + *a*, where *m* indicates the growth parameter, *m* = 1 and 2 describe interface-controlled growth or volume diffusion-controlled growth, respectively; *d* = 1, 2, 3 stands for the dimensionality of the growth [33]) can be used to cover the prevailing mechanism, i.e., the isokinetic assumption holds for the above transformations. For example, if *d*/*m* = 3 and *a* = 1, the prevailing mechanism changes between a mixture of site saturation and interface-controlled growth (*d*/*m* = 3/1) and a combination of continuous nucleation and this growth mechanism; if *d*/*m* = 1.5 and *a* = 3, the prevailing mechanism changes between a mixture of site saturation and volume diffusion-controlled growth (*d*/*m* = 3/2) and a combination of continuous nucleation with increasing nucleation rate and volume diffusion-controlled growth. The constant *a* implies that the continuous nucleation follows an analogous increasing tendency, independent of the heating rate.

Now, a question arises about the transformations if the value of *a* changes with the heating rate. It was found that, for isochronal transformations conducted with a wide range of heating rates, the enthalpy difference curves recorded by differential scanning calorimetry (DSC) change abruptly. The Avrami exponent does not change as assumed by the analytical model (*d*/*m* < *n* < *d*/*m* + *a* with a single constant *a* [34]), which cannot describe the prevailing mechanisms. The crystallization DSC curves of some Fe-B amorphous alloys belong to this kind of transformation. In the present work, arising from the analytical model, an analytical approach based on the non-isokinetic analysis is proposed to study the crystallization kinetics of amorphous Fe_85_B_15_ alloy using isochronal annealing and DSC simultaneously.

## 2. Theoretical Background

### 2.1. Transformation Rate

Based on the analysis transformation model [15,16,17,18,19,20,21,22,23,24], for isochronal transformations with *n*, *Q* and *K*_0_ as constants (i.e., not affected by temperature *T*), the transformation rate *df*/*dT* follows [35]:

(1) $$\frac{df}{dT}=\frac{1}{\Phi }\frac{df}{dt}=\left(\frac{nQ}{RT^{2}}+\frac{2n}{T}\right)Ix_{e}$$

where *f* stands for the real transformed fraction, *R* means the gas constant, *x_e_* represents the extended transformed fraction and *I* stands for the impingement mode [35]. The function between *f* and *x_e_* depends on the impingement mode [19,35]. In comparison to the case with random nuclei dispersion (*I* = *df*/*dx_e_* = 1 − *f*) [4,19], the impingement induced by anisotropic growth is more intense with *I* = (1 − *f*)^ξ^ [19,35], whereas the impingement according to non-randomly dispersed nuclei is less strong with *I* = (1 − *f*^ε^) [19,35]. Both *ξ* and *ε* are constant factors larger than 1.

Usually, the condition *2n*/*T* << *nQ*/*RT*^2^ always holds, hence the set *2n*/*T* can be neglected in Equation (1) [33]. Then, it follows that

(2) $$\frac{df}{dT}=\frac{1}{\Phi }\frac{df}{dt}=\frac{nQ}{RT^{2}}Ix_{e}$$

where *t* represents the time and Φ stands for the heating rate.

Following the analytical model, for an isochronal transformation, its rate can be obtained by [35]:

(3) dfdT=1Φdfdt=nQΦI(f)[xe(f)]1−1nK0exp(−QRT)

Explicit expressions for *x_e_*, *n*, *Q* and *Ι* are provided in Tables 1 and 2 in Reference [19].

### 2.2. Non-Isokinetic Analysis

Generally, the transformed fraction *f* and the transformation rate *df*/*dT* can be directly obtained from DSC measurements. Applying the maximum peak analysis for a single DSC curve yields information on the impingement [35]. The application of Equation (2) to the DSC curve directly yields the value of *nQ*. Section 4.3 shows that the isokinetic approach to deduce *nQ* is the prerequisite for the subsequent non-isokinetic analysis. For other methods to deduce *nQ*, see Appendix A.

An equivalent, constant growth exponent *n* holds for every single transformation and is given according to the following equation [36,37,38,39] (irrespective of whether it proceeds according to continuous, site saturation or mixed nucleation mechanisms [18]):

(4) $$n=\frac{d}{m}+a$$

Therefore, for a series of isochronal transformations, the value of *a* described by non-isokinetics changes with the heating rate.

For any typical nucleation–growth transformation, the following equation holds [24]:

(5) $$Q=\frac{\frac{d}{m}\cdot Q_{G}+\left(n-\frac{d}{m}\right)\cdot Q_{N}}{n}\;=\frac{\frac{d}{m}\cdot Q_{G}+aQ_{N}}{n}$$

where *Q_N_* and *Q_G_* represent the activation energy for nucleation and growth. Then it follows that

(6) $$nQ=\frac{d}{m}Q_{G}+\left(n-\frac{d}{m}\right)Q_{N}$$

On this basis, combination with Equations (3) and (5) leads to

(7) dfdt=dfdT·Φ=nQI(f)[xe(f)]1−1nK0exp(−QRT)=[dmQG+(n−dm)QN]I(f)[xe(f)]1−1nK0exp(−QRT)

The kinetic parameters (*n*, *Q* and *K*_0_), as well as *Q_N_* and *Q_G_*, can be obtained by fitting of Equation (7) to a series of isochronal DSC curves, in combination with the *nQ* values deduced from Equation (2), (see Section 4.3).

## 3. Materials and Methods

Fe_85_B_15_ master alloys were obtained by the induction melting of bulk high-purity iron and an inter-alloy of iron-boron under a protective argon atmosphere. A melt spinning technique with argon atmosphere was used to prepare amorphous ribbons. The thickness of the ribbons was about 32 µm. X-ray diffraction (XRD) (Panalytical, Almelo, The Netherlands) and transmission electron microscope (TEM) (Tecnai F30 G^2^, FEI Company, Hillsboro, OR, United States) were applied to verify the amorphous nature of the prepared samples. Inductively coupled plasma (ICP) optical emission spectrometry technique was utilized to test the composition of the resulting ribbons; the chemical composition of the as-prepared ribbons was 96.5 ± 0.2 wt.% Fe and 3.5 ± 0.2 wt.% B, and the weight composition of Fe_85_B_15_ alloy comprised 96.7 wt.% Fe and 3.3 wt.% B.

Differential scanning calorimeter (DSC) measurements were performed during the annealing of the amorphous alloys on a power compensated Perkin Elmer DSC-7 (PerkinElmer Inc., Waltham, MA USA). The DSC instrument with alumina sample pans and covers works under flowing high-purity argon (99.995%). Pure In, Sn, Bi, Zn, Al and Au specimens with a weight of 10 mg were used to calibrate the DSC. The melting temperatures and the heat of fusion of the pure samples were measured for the temperature and the heat flow calibration. Due to the small weight (about 3 mg) of the ribbons during annealing, an empty alumina pan with a cover was used as a reference.

The ribbons were annealed isochronally using different heating rates of 5, 10, 20, 30 and 40 K min^−1^. The annealing at each heating rate was performed twice. During the first run, the crystallization transformation from amorphous to crystalline of the sample occurs. The second run shows whether the crystallization was completely accomplished and is further used as baseline. The curve of the transformation’s enthalpy change is obtained by subtracting the second run from the first run.

Phase analysis was accomplished on an X’pert Pro MRD diffractometer (Co-Kα radiation, Panalytical, Almelo, The Netherlands) between 30° and 120° (2θ value) with an interval of 0.04°. The preparation of TEM samples was performed using argon ion milling on a Gatan model 691 with an incidence angle of 13.5° and 3.5 kV acceleration voltage. The investigation of the microstructure of the annealed samples was carried out by applying a Tecnai F30 G^2^ TEM with an accelerating voltage of 140 kV.

## 4. Results and Discussion

### 4.1. Amorphous Structure

**Figure 1:**
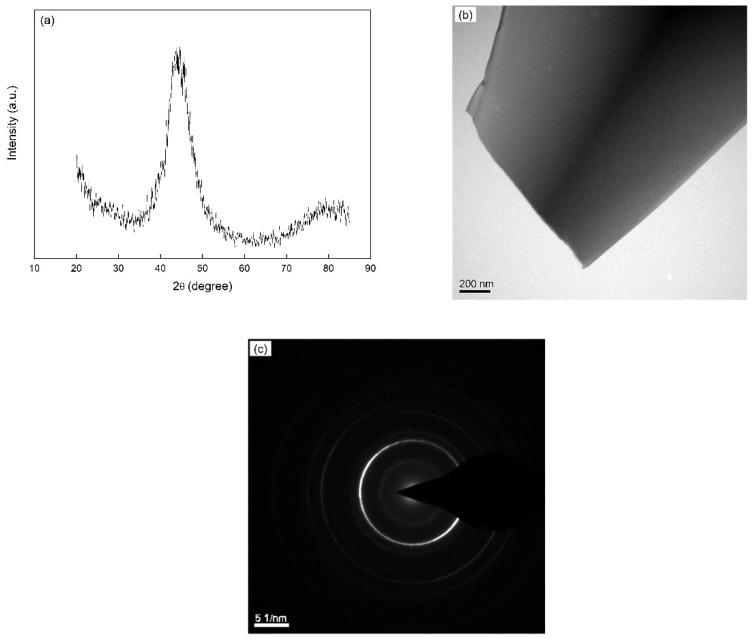
(**a**) X-ray diffraction pattern (Co-Kα radiation), (**b**) TEM bright field image and (**c**) the corresponding selected area electron diffraction pattern (SAEDP) of the amorphous as-prepared Fe_85_B_15_ alloy. The XRD and TEM investigations were carried out at room temperature.

XRD was performed to verify the amorphous structure and determine the crystallization product. The side of the ribbons in contact with the copper roller appears dull while the opposite (free) side is shiny. However, both the dull and the shiny side show the same XRD diffraction patterns (Figure 1a). A broad pattern can be observed in the X-ray diffractogram. The as-prepared ribbon was examined by TEM. The bright field image (Figure 1b) and the selected area electron diffraction pattern (SAEDP, Figure 1c) evidence that the as-quenched structure is entirely amorphous.

### 4.2. Crystallization Product

**Figure 2:**
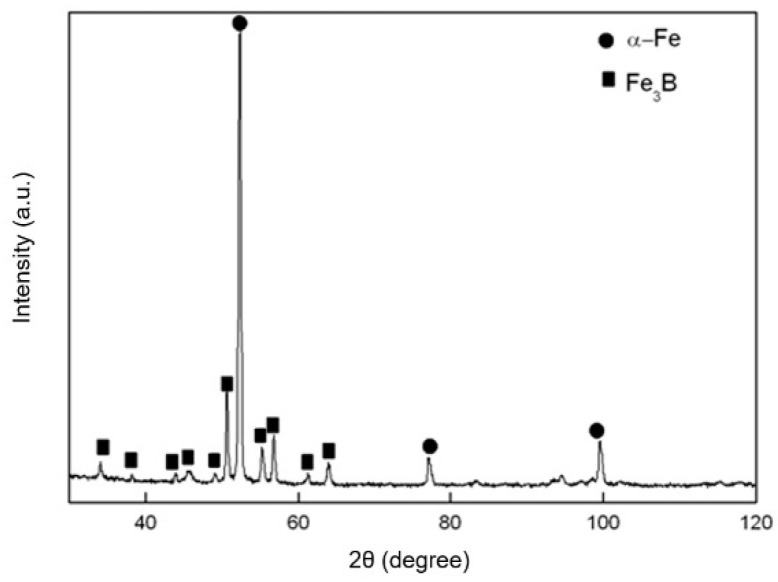
XRD profile for the crystallized Fe_85_B_15_ alloy annealed at a rate 5 K min^−1^. The crystalline in the alloy was detected as α-Fe (PDF number: 06-0696) and tetragonal Fe_3_B (PDF number: 39-1315) by applying the MDI Jade 6.0 program.

The sample annealed with a heating rate of 5 K min^−1^ in the DSC equipment was studied using XRD, and two phases, α-Fe (Powder Diffraction File, i.e., PDF with the number 06–0696) and tetragonal Fe_3_B (PDF: 39-1315), can be found in the X-ray diffractogram (Figure 2). The phase detection was performed utilizing MDI Jade 6.0 software. The result of the crystallization product is comparable with that in [40,41], in which α-Fe and tetragonal Fe_3_B were reported for Fe-B alloys with the boron composition between 14 at.% and 25 at.%.

**Figure 3:**
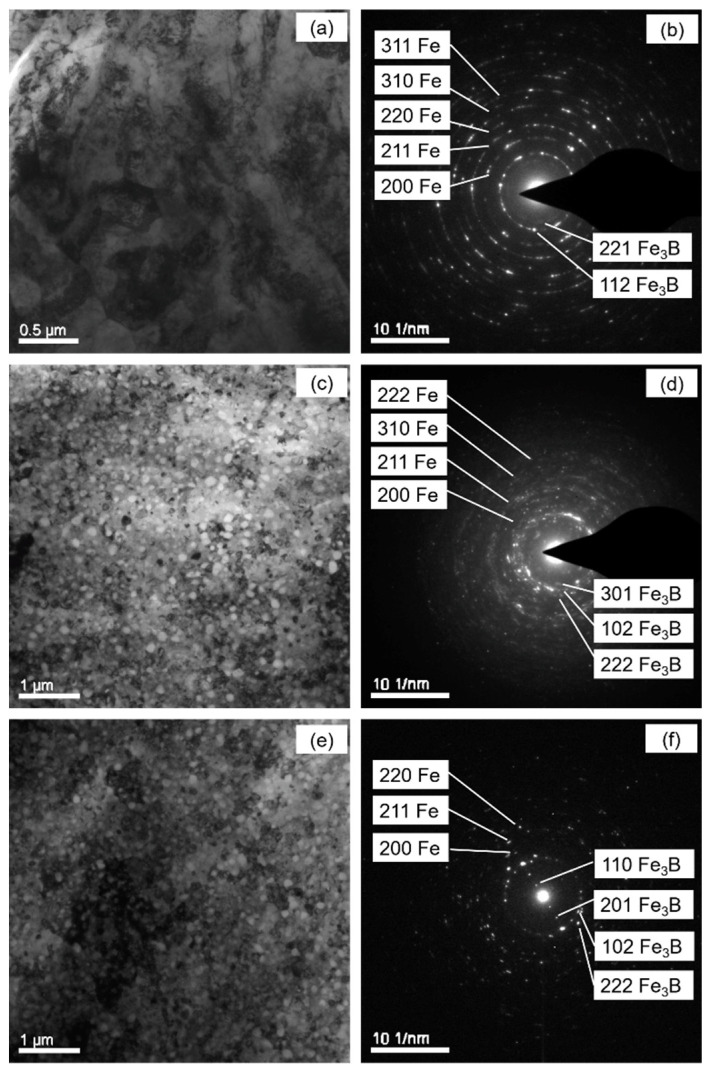
Bright field micrograph of TEM and SAEDPs of the crystallized Fe_85_B_15_ amorphous alloy after heated at 5 K min^−1^ ((**a**) and (**b**)); at 20 K min^−1^ ((**c**) and (**d**)) and at 40 K min^−1^ ((**e**) and (**f**)). The diameter for α-Fe (bcc) and tetragonal Fe_3_B phases have been identified in the SAEDPs (**b**,**d**,**f**).

The samples annealed with Φ = 5, 20 and 40 K min^−1^ were investigated using TEM (Figure 3). Nano-scaled grains with an approximately globular shape can be observed in the TEM bright field images (Figure 3a,c,e). In the corresponding SAEDPs (Figure 3b,d,f), numerous spots are present and aligned in circles because of the characteristic of the nanosized microstructure. The diameter of the circles with diffraction spots can be assigned to the α-Fe and tetragonal Fe_3_B phases, which correspond to the XRD result. The particles in the crystallized specimens observed in the bright field images (Figure 3a,c,e) contain two phases, i.e., pure particles of either α-Fe phase or Fe_3_B phase cannot be found.

### 4.3. Crystallization Kinetics

**Figure 4:**
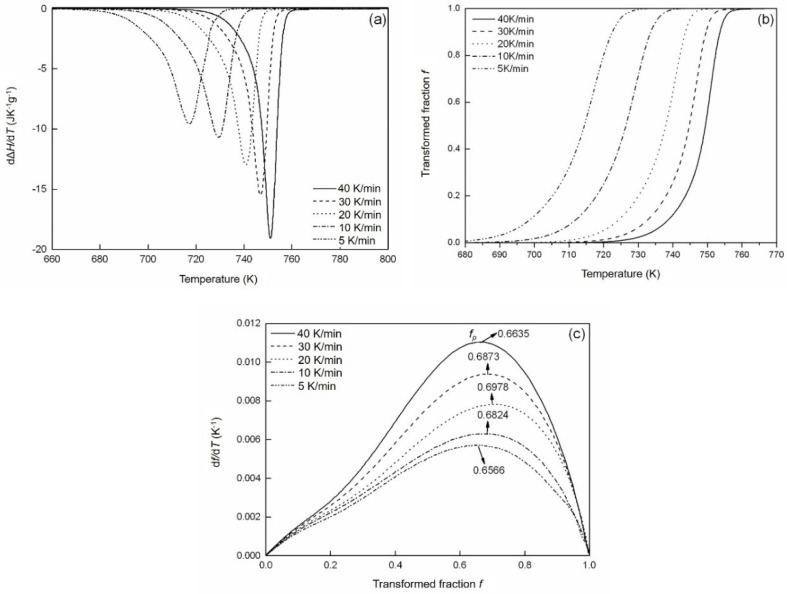
(**a**) Rate of enthalpy change divided by the heating rate *d*Δ*H*/Φ*dt* = *d*Δ*H*/*dT*, (**b**) evolution of transformed fraction *f* with temperature *T* and (**c**) evolution of *df*/*dT* (transformation rate) with transformed fraction *f*, derived from differential scanning calorimetry (DSC) measurement of the amorphous Fe_85_B_15_ alloy after being isochronally crystallized, with heating rates Φ of 5, 10, 20, 30 and 40 K min^−1^. The values of *f_p_* are larger than 1 − 1/e (**c**).

The isochronal DSC scans for the crystallization of amorphous Fe_85_B_15_ ribbons were recorded at different heating rates of 5, 10, 20, 30 and 40 K min^−1^. One single exothermic peak appears in the DSC measurements due to the crystallization reaction (Figure 4a). The XRD and TEM analyses show that two phases are present after the crystallization. Therefore, the two phases are formed in one step during the crystallization of Fe_85_B_15_ alloy, in which both phases grow simultaneously. The rate of the enthalpy difference *d*Δ*H*/*dt* during the crystallization can be obtained after performing the baseline correction [42], in which Δ*H* represents the enthalpy difference in crystallization with a negative value. The transformed fraction *f* is defined as the ratio of the enthalpy change Δ*H* to the total crystallization enthalpy Δ*H_tot_*, as determined from the DSC experiments, i.e., *f* = Δ*H*/Δ*H_tot_*. The baseline-corrected isochronal DSC scans are depicted in Figure 4a. The averaged total crystallization enthalpy subjected to different heating rates is about 168 ± 3 J g^−1^ according to Figure 4a, which is in agreement with the data in [43,44]. The obtained transformed fraction *f* vs. temperature is reproduced in Figure 4b. In Figure 4c, the evolution of the transformation rate *df*/*dT* with *f* is represented.

**Figure 5:**
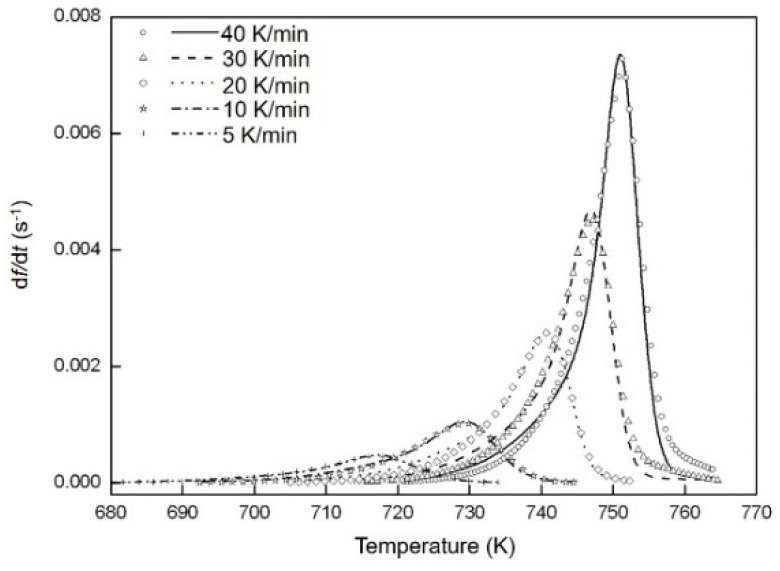
The transformation rate due to isochronal crystallization of amorphous Fe_85_B_15_ alloy at the heating rates indicated, as measured by DSC (lines), and as fitted by the current model (symbols). The fitting result from Equation (7) of heating rates 5, 10, 20, 30 and 40 K min^−1^. The fitting parameters obtained are given in Table 1.

The temperature at the peak of the transformation rate increases with increasing Φ, accompanied by a simultaneous increase in the maximum transformation rate (Figure 4c and Figure 5). This observation indicates that the transformation follows non-isokinetics (i.e., abnormal growth exponent occurs upon increasing Φ; see Section 2.2). Otherwise, the transformation rate according to Equation (2) would decrease monotonically with increasing Φ [15,19]. The kinetic parameters *n*, *Q* and *K*_0_, as well as the impingement mode, are obtained by fitting the isochronally conducted transformations by utilizing Equation (7).

For isochronal transformations, the transformed fraction at the peak of the transformation rate, i.e., *f_p_* does not depend on the value of *n* [35]. As exhibited in Figure 4c, the impingement according to non-random dispersed nuclei (with the impingement factor *ε* > 1 [28]) results from *f_p_* > 1 − 1/e [28]. In the current mode, *ε* is chosen as a fitting parameter in the fitting of the transformation curves.

As shown in Section 2.2, for every single transformation, the product of *nQ* can be calculated according to Equation (A7) with the experimental DSC data points at the peak maximum of the transformation curves, about 618.33, 730.86, 932.20, 1236.25 and 1836.54 kJ mol^−1^, respectively, for heating rates from 5 K min^−1^ to 40 K min^−1^. Using Equation (7) and the impingement mode deduced above, the evolution of *df*/*dt* according to temperature *T* can be calculated.

**Figure 6:**
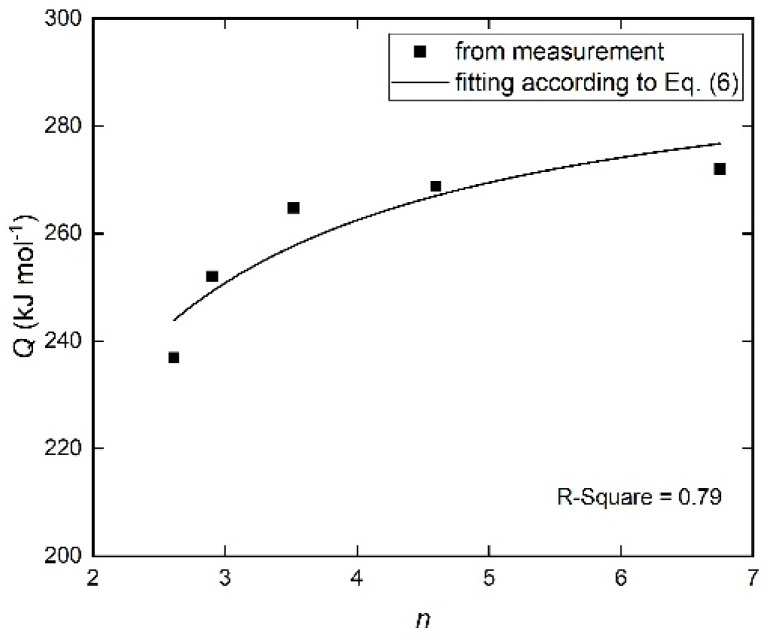
The evolution of the overall effective activation energy *Q* and the Avrami (growth) exponent *n* with heating rates of 5–40 K min^−1^. The separate activation energy for nucleation and growth is obtained by fitting Equation (6) to the data points with *n* and *Q* values listed in Table 1. The result is *Q_N_* = 297 ± 10 kJ mol^−1^, *Q_G_* = 204 ± 33 kJ mol^−1^, with an R-square value of about 0.79.

The fitting was carried out by applying a downhill simplex method [45]. With adjusting the kinetic parameters *K*_0_, *n* and *ε*, the least-squares difference is obtained between the calculated values using the model and the experimentally measured curves. The quality of the fitting is given by the ratio between the total of the absolute differences (i.e., fitted *df*/*dt* values subtracted from the measured *df*/*dt* values) and the sum of all the fitted *df*/*dt* absolute values. The fitting results are reproduced in Figure 5. The kinetic parameters *K*_0_, *n* and *ε* determined by this analysis are summarized in Table 1. The overall activation energy *Q* at each heating rate can then be calculated from the values of *nQ*; the activation energies for nucleation *Q_N_* and growth *Q_G_* can be obtained according to Equation (6) (Figure 6).

As shown in Table 1, the growth exponent *n* is fitted as values increasing from ~2.6 to ~6.8 with increasing Φ, thus indicating that the transformation follows non-isokinetics (i.e., *a* is accordingly changing with the heating rate). In combination with Section 4.2, the crystallization is therefore controlled by mixed nucleation with the nucleation index *a*, volume diffusion-controlled growth in three dimensions (i.e., *d* = 3 and *m* = 2; see Equation (7)) and impingement mode according to non-randomly dispersed nuclei. This is supported by the microstructure after crystallization (Figure 3). The growth mode corresponds to the formation of two new phases from the amorphous matrix during crystallization (Section 4.2).

As shown in Figure 6, the activation energies for nucleation *Q_N_* and growth *Q_G_* during the transformation are deduced as 297 ± 10 kJ mol^−1^ and 204 ± 33 kJ mol^−1^, respectively. These data can be compared with the diffusion data of crystalline iron-boron alloys: the activation energy for boron diffusion in α-iron provided by Busby et al. [46,47,48] is *Q* = 260 kJ mol^−1^; the activation energy for self-diffusion of Fe in α-iron is *Q* ≈ 239.7 kJ mol^−1^ [49]. Accordingly, the obtained result for *Q_G_* is reasonable. The value for *Q_N_* (297 ± 10 kJ mol^−1^) is almost the same as the nucleation activation energy values obtained for metal-metalloid glasses (Fe_65_Ni_10_B_25_: 300 kJ mol^−1^ [50]). The result for *Q_N_* can be therefore considered plausible as well.

**Figure 7:**
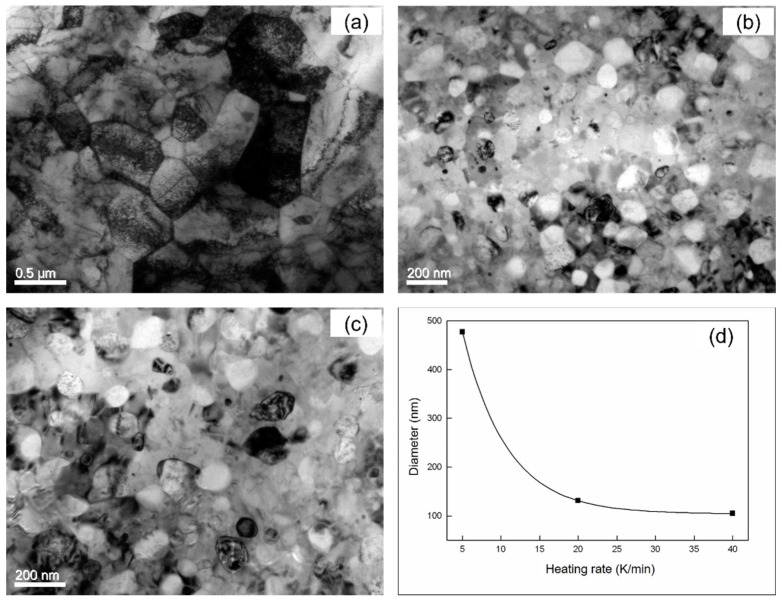
Bright field micrograph of TEM of the crystallized Fe_85_B_15_ alloy annealed at the heating rates of 5 K min^−1^ (**a**), 20 K min^−1^ (**b**) and 40 K min^−1^ (**c**). (**d**) The average crystal size after crystallization at the heating rates of 5, 20 and 40 K min^−1^ has been calculated with linear intercept method accordingly (symbols). The line is drawn as a guide to the eye.

The crystalline after crystallizations with Φ = 5, 20, 40 K min^−1^ are represented in Figure 7a–c. The average grain size of the crystalline can be calculated using the linear intercept method (Figure 7d). Its diameter decreases from ~400 nm to ~100 nm when Φ increases from 5 K min^−1^ to 40 K min^−1^, suggesting a strong heating rate dependence of the as-crystallized grain size. This is compatible with the acceleration of the nucleation rate because of a progressing transformation during isochronal crystallization (*a* = 5.25 for Φ = 40 K min^−1^ and *a* = 1.11 for Φ = 5 K min^−1^). The higher value of the index *a* indicates the highly increased nucleation rate upon crystallization with a higher heating rate.

## 5. Conclusions

A non-isokinetic approach has been performed to study transformations with abrupt changing of DSC peaks for isochronally conducted transformation. Accordingly, the growth exponent *n* and the effective activation energy *Q* of a series of isochronal transformations can be quantitatively described. The following conclusions can be drawn:

(1) A different analytical model based on non-isokinetics was proposed to describe real transformations with a growth exponent *d*/*m* < *n* < *d*/*m* + *a*. As such, the kinetic parameters controlling crystallization of amorphous Fe_85_B_15_ alloys were obtained. These are compatible with a decreasing average grain size with increasing heating rates, because of the increasing nucleation rate.

(2) The isochronal crystallization of the Fe_85_B_15_ amorphous alloy is controlled by mixed nucleation including nucleation index, *a*, three-dimensional volume diffusion-controlled growth (i.e., *d* = 3 and *m* = 2) and impingement mode due to non-randomly dispersed nuclei.

(3) The isochronally crystallized product was investigated by applying XRD and TEM. The nanosized particles contain two phases, i.e., α-Fe and Fe_3_B.

(4) By applying the current analytical model to the isochronal crystallization of Fe_85_B_15_, reasonable values for the activation energies of nucleation (*Q_N_* = 297 ± 10 kJ mol^−1^) and growth (*Q_G_* = 204 ± 33 kJ mol^−1^) were obtained.

## Figures and Tables

**Table 1:** Kinetic parameters *n*, *Q*, *K*_0_, nucleation index *a* and impingement factor *ε*, as determined by fitting the measured DSC curves using the current model for the isochronally crystallized amorphous Fe_85_B_15_ alloy (see Section 4.3), with the initial assumed values *n* = 4, *K*_0_ = 1 × 10^9^ s^−1^, *Q* = 300 kJ mol^−1^ during fitting.

Φ (K min^−1^)	*n*	*Q* (kJ mol^−1^)	*K*_0_ (s^−1^)	*ε*	*a*	*Error* (%)
5	2.61	236.9	2.8 × 10^8^	1.88	1.11	4.68
10	2.90	252.0	3.1 × 10^9^	2.23	1.40	1.83
20	3.52	264.8	2.2 × 10^10^	3.72	2.02	6.61
30	4.60	268.8	4.2 × 10^10^	3.28	3.08	4.81
40	6.75	272.1	6.1 × 10^10^	2.75	5.25	2.74

## Data Availability

The data presented in this study are available on request from the corresponding author. The data are not publicly available at this time due to the data also forms part of an ongoing study.

## Appendix A

Generally, the peak of the DSC curve corresponds to the maximum transformation rate, i.e., *d*^2^*f*/*dT*^2^ = 0. When the impingement mode is according to random nuclei dispersion, the following condition must be satisfied at the peak [35]:

(A1) $$0=\left[1+\ln\left(1-f_{p}\right)\right]$$

Assume the temperature of peak maximum as *T_p_*, then [35]

(A2) $$\frac{\Phi }{T_{p}^{2}}=RK_{0}\exp\left(-\frac{Q_{p}}{RT_{p}}\right)$$

Given $K=K_{0}\exp\left(-\frac{Q}{RT}\right)$, then this results in $K_{p}=K_{0}\exp\left(-\frac{Q_{p}}{RT_{p}}\right)$.

Subsequently,

(A3) $$\frac{K}{K_{p}}=\exp\left(\frac{TQ_{p}-T_{p}Q}{RTT_{p}}\right)$$

where $\frac{K}{K_{p}}≅\exp\left(\frac{Q}{RT_{p}^{2}}\left(T-T_{p}\right)\right)$ approximately.

Following [51], where the basic assumptions (*T* − *T_p_* = Φ*t* and $\frac{K}{K_{p}}=\exp\left(\frac{Q}{R}\frac{\Phi t}{T_{p}^{2}}\right)$) are made, this results in

(A4) $$K=K_{0}\exp\left(-\frac{Q_{p}}{RT_{p}}\right)\exp\left(QK_{0}\exp\left(-\frac{Q_{p}}{RT}\right)t\right)$$

Assuming $t^{\prime }=t\left[\exp\left(-\frac{Q_{p}}{RT}\right)\right]$ leads to

(A5) dxedt′=nQK0[xe]1−1nexp(QK0t′)

with $K_{0}=\exp\left(\frac{Q_{p}}{RT_{p}}\right)\frac{\Phi }{RT_{p}^{2}}$.

As compared to *t′*, for the case with the impingement mode according to random nuclei dispersion and isokinetic assumption, i.e., *Q* = *Q_p_*,

(A6) $$f=1-\exp[-{\left[\exp\left(\left(\frac{Q}{RT_{p}^{2}}\right)\left(T-T_{p}\right)\right)\right]}^{n}]$$

From (A6), this also results as

(A7) ∂ln(−ln(1−f))∂(T−TpTp2)=nQR
